# The CROWN Initiative: journal editors invite researchers to develop core outcomes in women’s health

**DOI:** 10.1186/s40834-016-0024-3

**Published:** 2016-09-13

**Authors:** Khalid Khan, On behalf of Chief Editors of Journals participating in The CROWN Initiative listed at the end of this article

**Affiliations:** RCOG, 27 Sussex Place, London, NW1 4RG UK

**Keywords:** Research design/standards, Treatment outcome, Endpoint determination/standards, Clinical trials, Systematic reviews, Guidelines, Bias (Epidemiology), Evidence-based medicine, Consensus

## Abstract

ᅟ

Clinical trials, systematic reviews and guidelines compare beneficial and non-beneficial outcomes following interventions. Often, however, various studies on a particular topic do not address the same outcomes, making it difficult to draw clinically useful conclusions when a group of studies is looked at as a whole [1]. This problem was recently thrown into sharp focus by a systematic review of interventions for preterm birth prevention, which found that among 103 randomised trials, no fewer than 72 different outcomes were reported [2]. There is a growing recognition among clinical researchers that this variability undermines consistent synthesis of the evidence, and that what is needed is an agreed standardised collection of outcomes – a “core outcomes set” – for all trials in a specific clinical area [1]. Recognising that the current inconsistency is a serious hindrance to progress in our specialty, the editors of over 50 journals related to women’s health have come together to support The CROWN (CoRe Outcomes in WomeN’s health) Initiative (Table 1).

**Table 1:** Aims of the CROWN initiative

1	Form a consortium among all gynaecology-obstetrics and related journals to promote core outcome sets in all areas of our specialty.
2	Encourage researchers to develop core outcome sets using robust consensus methodology involving multiple stakeholders, including patients.
3	Strongly encourage the reporting of results for core outcome sets.
4	Organise robust peer-review and effective dissemination of manuscripts describing core outcome sets.
5	Facilitate embedding of core outcome sets in research practice, working closely with researchers, reviewers, funders and guideline makers. www.crown-initiative.org

Development of consensus is required around a set of well-defined, relevant and feasible outcomes for all trials concerning particular obstetric and gynaecologic health conditions, such as preterm birth, incontinence, infertility and menstrual problems. With so many subspecialties involved, this is no easy task. Duplication of effort can be avoided by working with the Core Outcome Measures in Effectiveness Trials (COMET) Initiative, which is working towards core data sets for all medical specialties [3]. Production of trustworthy core outcome sets will require engagement with patients, healthcare professionals, researchers, industry and regulators, and the employment of scientifically robust consensus methods [1]. The data for these core outcome sets, once agreed upon, should be collected in trials and reported in publications as standard practice in the future.

Journal editors now invite researchers to take the lead in beginning this work. What will we do as editors to support them and their colleagues? First, we are drawing wide attention to The CROWN Initiative by publishing this editorial in the journals listed below. We shall ensure that the global research community, which includes our many reviewers, is aware of the need for core outcome sets. Submissions which describe development of core outcome sets, if deemed acceptable after peer review, will be effectively disseminated.

Our collaboration is not for enforcing harmony at the expense of innovation. To quote from the COMET home page (www.comet-initiative.org): “The existence or use of a core outcome set does not imply that outcomes in a particular trial should be restricted to those in the relevant core outcome set. Rather, there is an expectation that the core outcomes will be collected and reported, making it easier for the results of trials to be compared, contrasted and combined as appropriate; while researchers continue to explore other outcomes as well.” We also expect that as new or superior ways of capturing outcomes emerge, core outcome sets will themselves need updating.

Producing, disseminating and implementing core outcome sets will ensure that critical and important outcomes with good measurement properties are incorporated and reported. We believe this is the next important step in advancing the usefulness of research, in informing readers, including guideline and policy developers, who are involved in decision-making, and in improving evidence-based practice.
